# The Effect of Stress, Anxiety and Depression on In Vitro Fertilization Outcome in Kazakhstani Public Clinical Setting: A Cross-Sectional Study

**DOI:** 10.3390/jcm10050937

**Published:** 2021-03-01

**Authors:** Gauri Bapayeva, Gulzhanat Aimagambetova, Alpamys Issanov, Sanja Terzic, Talshyn Ukybassova, Aidana Aldiyarova, Gulnara Utepova, Zhanibek Daribay, Gulnara Bekbossinova, Askhat Balykov, Antonio Simone Laganà, Milan Terzic

**Affiliations:** 1 Clinical Academic Department of Women’s Health, National Research Center of Mother and Child Health, University Medical Center, Nur-Sultan 010000, Kazakhstan; gauri.bapayeva@gmail.com (G.B.); talshynu@yandex.ru (T.U.); Aidana.aldiyarova@gmail.com (A.A.); gulnara.utepova@umc.org.kz (G.U.); milan.terzic@nu.edu.kz (M.T.); 2 Department of Biomedical Sciences, Nazarbayev University School of Medicine, Nur-Sultan 010000, Kazakhstan; 3 Department of Medicine, Nazarbayev University School of Medicine, Nur-Sultan 010000, Kazakhstan; alpamys.issanov@nu.edu.kz (A.I.); sanja.terzic@nu.edu.kz (S.T.); 4 Department of Obstetrics and Gynecology #2, Marat Ospanov West Kazakhstan State Medical University, Aktobe 030000, Kazakhstan; zhanibek.daribay@gmail.com; 5 Regional Perinatal Center, Aktobe 030000, Kazakhstan; kdo@aktobe-opc.kz (G.B.); balykov.askhat@gmail.com (A.B.); 6 Department of Obstetrics and Gynecology, “Filippo Del Ponte” Hospital, University of Insubria, 21100 Varese, Italy; antoniosimone.lagana@uninsubria.it; 7 Department of Obstetrics, Gynecology and Reproductive Sciences, University of Pittsburgh School of Medicine, Pittsburgh, PA 15213, USA

**Keywords:** infertility, in vitro fertilization, stress, depression, anxiety, Kazakhstan

## Abstract

Although it is clear that infertility leads to heightened stress for patients, the impact of depressed mood and anxiety on treatment outcome is inconsistently reported. The aim of this study was to evaluate the effect of stress, depression and anxiety on in vitro fertilization (IVF) outcomes in Kazakhstani public assisted reproductive technology (ART) clinics. The prospective cohort study was performed between June 2019 and September 2020 using questionnaires to assess psychological stress, depressed mood and anxiety in women referred to IVF clinics in two public clinical centers in Kazakhstan, Nur-Sultan and Aktobe. Our study sample comprised 142 women with the average age of 33.9 ± 4.9 years, and infertility duration 6.0 ± 3.5 years. More than half of respondents had Center for Epidemiological Studies Depression Scale (CES-D) scores higher than 16, indicating their risk of developing clinical depression. Ninety-one percent of women from Aktobe city were at risk for clinical depression (*p* < 0.001). Aktobe city respondents had higher stress subscale scores and anxiety scale scores (*p* < 0.001) than Nur-Sultan respondents. Statistical analysis showed that IVF outcome was not significantly associated with depression and stress, while the higher anxiety scale scores were negatively associated with clinical pregnancy after IVF.

## 1. Introduction

Infertility is defined as failure to achieve pregnancy within 12 months of unprotected intercourse or therapeutic donor insemination in women younger than 35 years or within 6 months in women older than 35 years [1,2]. Despite huge progress in the development of artificial reproductive technologies (ART) in the 21st century, infertility remains a highly prevalent global problem. Furthermore, industrial development, urbanization, and global trends in lifestyle changes may be contributing to a rise in fertility problems [3]. Infertility is estimated to affect 8–15% of reproductive-aged couples worldwide [1,4]. The rates are different in high and low-income countries. In some developing regions of the world, the rates of infertility are much higher, reaching 30% in some populations [5,6,7]. Due to high prevalence of infertility, it has been highlighted as a social disease by the World Health Organization (WHO) [6,7]. 

Many people associate being female with the ability to conceive and bear a child. Thus, infertility can leave a woman feeling different, defective, or out of step with her peers. Infertility can also disrupt a woman’s life goals and result in loneliness, powerlessness, and stigmatization [8,9]. Women who experience infertility report elevated levels of stress, anxiety and depression. However, the impact of depressed mood and anxiety on infertility treatment outcome remains unclear [10,11,12]. The effect of facing fertility problems on couple relationships is highly varied, with some experiencing increased cohesion and others becoming distanced from each other and even splitting apart [10,12,13]. Recent research has revealed 25–35% of couples report that the experience brought them closer together or strengthened their relationship [10,14]. A precise cause-effect relationship is still inconsistently reported due to conflicting results and the different evaluation tools/surveys employed [11,12]. Therefore, further investigations in this area are necessary.

Kazakhstan is one of the post-soviet Central Asian republics, which achieved independence in 1991. According to the data from 2018, the Kazakhstani population consisted of more than 18 million people with 26.7% of women being of fertile age [15]. The population of the country is diverse and consists of many ethno-cultural groups. The biggest ethnic group is Kazakh ethnicity, which has long-term traditions of family formation with specific focus on a culture that promotes having children [16,17]. Even in the 21st century, women’s fertility determines her status in the traditional Kazakh family society [17]. Thus, being able to conceive and have children are central life goals for Kazakhstani women. 

In Central and Eastern European and Central Asian regions, countries report high rates of infertility [18]. In the Republic of Kazakhstan the frequency of infertile couples varies from 12% to 15.5% [3,15]. 

In vitro fertilization (IVF) has been used in clinical practice for more than 40 years [4]. However, IVF remains unavailable for the majority of the world’s infertile couples [4,5]. The lack of access to IVF clinics in some countries and the high cost of IVF in many others have pushed some governments to develop national support programs [4,5]. Kazakhstan has universal health care coverage and free access to medical procedures. The first private IVF laboratory in Kazakhstan was opened in 1995 with the first newborn delivered in 1996. Since 2010, the Kazakhstani Ministry of Healthcare has provided funds for IVF coverage and a few public IVF clinics have been established. Although the funds are limited in amount, for the period of 2010–2018 with governmental support around 3000 IVF-conceived babies were born. 

According to the Kazakhstani Association of Reproductive Medicine (KARM), the demand for IVF treatment is high due to a growing prevalence of infertility. It is estimated that between 12,000 and 13,000 IVF procedures should be covered annually by the government to ensure that everyone who requires infertility management with IVF receives treatment [19]. As was announced by the Kazakhstani Ministry of Healthcare, the number of IVF procedures supported by the government, will be increased in 2021 to 7000 IVF cycles. This is seven times more than in 2020 where only 1000 IVF cycles were covered from the state budget.

Taking into consideration the above mentioned factors influencing IVF procedures and results, the aim of this study was to assess the effect of stress, depression and anxiety on IVF outcomes in Kazakhstani public IVF clinics.

## 2. Materials and Methods

### 2.1. Study Design

The prospective cohort study was performed from June 2019 to September 2020 in two public ART clinics in Kazakhstan including the National Research Center for Mother and Child Health (ART department) located in Nur-Sultan and the Regional Perinatal Center (ART department) located in Aktobe. Nur-Sultan is the capital city with a population size of 1 million people and Aktobe is the largest industrial city in the western part of the country with a population of about 0.3 million people. Women referred for initial or repeated IVF treatment at the ART clinics were provided with oral and written information about the study and asked to participate. All participants met the following inclusion criteria: (1) seeking IVF, (2) ≥18 years old, (3) able to answer questions in Kazakh, Russian, or English. Exclusion criteria were: (1) not able to answer questions in Kazakh, Russian, or English; (2) younger than 18 years old; (3) refusal to participate. This study was approved by the Institutional Research Ethics Committee of Nazarbayev University (protocol code 120/28012019, date of approval 13 March 2019). Written informed consent was obtained from each participant.

### 2.2. Data Collection

The eligible respondents were invited to participate in the study after careful explanation of the research importance for the country and the value of the research results for the local healthcare system. Any patient who agreed to participate was asked to sign an informed consent form and complete the questionnaires. Clinical pregnancy, defined as a live intrauterine pregnancy identified by ultrasound scan at eight weeks gestation, was the outcome variable. Patients, who had not yet reached 8 weeks gestation, had not yet had an ultrasound to determine the presence or absence of a clinical pregnancy. Consequently, these patients’ outcome data were categorized as “unknown”. The baseline questionnaire collected data about socio-demographic characteristics, such as age, body mass index (BMI), and education level. Education level was categorized according to International Standard Classification of Education (ISCED 4—secondary high school, ISCED 5—post-secondary non-tertiary education and ISCED 6—bachelor/master level education). The patients’ past medical history was collected using standardized survey. Specific information collected included all comorbidities associated with infertility, duration of infertility, number of previous deliveries, number of previous miscarriages, number of intentional pregnancy interruptions and number of previous IVF cycles performed. Further information collected included the number of oocytes retrieved, the number of embryos transferred, the cause of infertility (female, male and mixed), the type of treatment protocol, fertilization and implantation rates. 

### 2.3. Measurements

Validated psychometric questionnaires were used to measure baseline depression, infertility-related stress, state and trait anxiety levels.

#### 2.3.1. CES-D Scale

Depression was measured using the Center for Epidemiological Studies Depression Scale (CES-D) [20]. The CES-D questionnaire was employed for the study, as it is one of the widely used clinical screening tools for the presence of depression symptoms among pregnant women and the general population [21]. The CES-D is a 20-item questionnaire that measures nine components of depression including sadness, loss of appetite, sleep, thinking/concentration, guilt/worthlessness, tiredness/fatigue, movement/agitation, and suicidal ideation. Each question assesses frequency of depressive symptoms experienced during the last week. The sum of all questions scores ranges from 0 to 60, where higher scores suggest a higher degree of depressed mood. Those who scored 16 or more were considered to be at risk of clinical depression. In this study, the Cronbach’s alpha coefficient for CES-D scale was 0.94.

#### 2.3.2. Fertility Problem Inventory Scale

To measure levels of infertility-related stress, the Fertility Problem Inventory (FPI) [22], a 46-item questionnaire, was used. FPI assesses five different aspects of infertility-related stress: social concerns, sexual concerns, relationship concerns, rejection of childfree life-style and need for parenthood. All of these FPI subscales contribute to the cumulative global infertility stress scores where a maximum sum of scores could be as high as 276 indicating the highest level of infertility-related stress. In this study, the Cronbach’s alpha coefficient for FPI global infertility stress scale was 0.88 (social concern = 0.73, sexual concern = 0.72, relationship concern = 0.72, rejection of childfree lifestyle = 0.85 and need for parenthood = 0.77). Correlation analysis indicated that the subscales of social concern, sexual concern and relationship concern had moderate correlation coefficients. 

#### 2.3.3. Spielberger State-Trait Anxiety Inventory Scale

Anxiety was evaluated with Spielberger State-Trait Anxiety Inventory (STAI) [23]. The STAI-S (state) anxiety scale consists of 20 items, each ranging in score from 1 to 4. The STAI-S scale measures how participants feel “right now, at the moment”. The STAI-T (trait) anxiety scale also consists of 20 statements that measures how respondents generally feel. Each anxiety scale score varies from a minimum of 20 to a maximum of 80. High scores indicate greater levels of anxiety. In this study, the Cronbach’s alpha coefficients for the STAI-S and the STAI-T subscales were 0.89 and 0.83, respectively. All scales were translated in Russian and Kazakh languages by experienced researchers and then translated back to check appropriateness to the original versions. Correlation analysis showed that the scales were appropriately measuring depression, stress and anxiety without overlapping areas.

### 2.4. Statistical Analysis

Statistical analysis was performed on STATA version 15 statistical software (StataCorp. 2017. Stata Statistical Software: Release 15. StataCorp LLC, College Station, TX, USA). Means and standard deviations, as well as medians and interquartile ranges were calculated for continuous variables while frequencies and percentages were used to describe categorical variables. To test relationships of the binary outcome variable with continuous exposure variables, independent student *t*-test or Wilcoxon rank-sum test were used, where appropriate. Associations with categorical independent variables were tested using chi-square test or Fisher’s exact test. The relationships of CES-D, FPI and STAI scales with clinical pregnancy were examined using simple and multiple negative binomial regression analysis. To determine “less biased” associations of each psychological condition with clinical pregnancy, the models were built to be parsimonious, including a reasonable number of covariates based on their statistical significance and clinical importance. The final multivariable models included women’s BMI, location, comorbidity, and number of previous IVF cycles. The models were tested for existence of multicollinearity, quazi-complete separation (fertilization rate and implantation rate), and interactions. A *p*-value of less than 0.05 was considered statistically significant.

## 3. Results

### 3.1. Study Sample

Overall, 142 women who underwent IVF participated in the study. The average age of the participants was 33.9 ± 4.9 years, and average infertility duration was 6.0 ± 3.5 years (Table 1).

More than one third of the women were overweight (37.2%, Table 1) and had education level at ISCED 4 or less (36.4%) and paid themselves (out-of-pocket) for IVF procedure (40.1%, Supplementary Table S1). More than half of the women had comorbidities (58.4%), while at least one in ten had a history of previous miscarriages or intentional pregnancy interruptions (Supplementary Table S2).

None of the women had IVF-related complications. Clinical pregnancy was confirmed in 35 out of 118 women while the clinical outcome of the IVF procedure was unknown for 24 women (17%).

No difference was detected between pregnant and not pregnant women in terms of age (*p* = 0.18), education level (*p* = 0.25), type of payment (*p* = 0.06), infertility duration (*p* = 0.57), number of previous deliveries (*p* = 0.09), number of previous miscarriages (*p* = 0.34), number of previous intentional pregnancy interruptions (*p* = 0.51), cause of infertility (*p* = 0.12), number of oocytes retrieved (*p* = 0.11), fertilization rate ((*p* = 0.63). Pregnancy rate was statistically significantly higher among women with normal or underweight BMI (*p* < 0.01), who lived in Aktobe city (*p* < 0.001), who had no history of comorbidities (*p* < 0.001), who had not previously attempted IVF cycle ((*p* = 0.05), who had higher mean number of embryos transferred (*p* = 0.01) and who used different treatment protocols (*p* = 0.05), (Supplementary Table S3). 

### 3.2. Depression, Stress and Anxiety

More than half of all respondents had a CES-D score higher than 16, indicating that they are at risk of developing clinical depression (Table 2).

Depression scale score was statistically significantly higher among pregnant women than not pregnant (21.9 ± 8.9 to 16.8 ± 9.8, respectively, *p* = 0.01). In the univariate regression analysis, one score increase in CES-D scale was associated with RR = 1.04 (1.00–1.07) of clinical pregnancy after IVF (Table 3). However, after adjustment for BMI, location, comorbidity, and number of previous IVF cycles, no statistically significant association (RR = 0.99, 95% CI 0.95–1.04) was found between depression scale score and IVF outcome in the multiple negative binomial regression analysis.

In the univariate analysis, no association was observed between FPI subscale scores and global stress score with the IVF outcome (Table 2). Similarly, in simple and multiple negative binomial regression analysis, all FPI subscale scores and global stress scale score were not associated with the IVF outcome (Table 3). 

In the univariate analysis, state and trait anxiety were not associated with clinical pregnancy (Table 2 and Table 3). However, in multiple negative binomial regression analysis, adjusting for BMI, location, comorbidity, and number of previous IVF cycles, higher anxiety scale scores were negatively associated with clinical pregnancy (Table 3). One-point increase in STAI State scale score was associated with 5% lower risk of clinical IVF pregnancy after controlling for the covariates, while additional one-point increase in STAI Trait scale was associated with 8% lower chance of successful IVF outcome.

## 4. Discussion

There are some countries such as France, Spain, and Israel that provide full coverage of IVF treatments as a matter of policy, while the others partially cover expenses (e.g., Portugal, Sweden, Turkey) [18]. Beginning in 2010, Kazakhstani health care system also started a support program by providing partial coverage for IVF treatment for infertile couples. A limited number of articles evaluating IVF outcomes in Kazakhstan have been published in the past [18,24,25]. However, to our knowledge this is the first study in Kazakhstan evaluating stress, anxiety and depression in infertile women and their effect on IVF outcome. 

In this study, the average age of patients was 33.9 years and the duration of infertility was 6.0 years. This is comparable with a study of infertility in Qatari women (32.5 and 5.0 years respectively), who follow the same religion and have a culture that promotes having children [26].

Our patients were not informed by the physician about the chance of getting pregnant after the IVF procedure. This approach eliminated patients’ awareness of the chance of getting pregnant after IVF and excluded the existence of factors such as “informational based optimism” or “informational based anxiety” that could influence the stress, anxiety or depression experienced by patients.

The main findings of the study suggests that more than half of the respondents had increased risk of developing clinical depression (*p* < 0.001), even though the expenses have been covered by the government. The respondents also had high FPI stress subscale scores and STAI anxiety scale scores. These findings are in line with our previously published findings from Kazakhstani private clinics [27]. It is also supported by other study results, which reported that patients who are struggling to conceive, experience depression, anxiety, isolation, and loss of control [11]. Levels of depression in patients with infertility have been compared with patients who have been diagnosed with cancer [11].

The stress, depression, and anxiety rates were higher in Aktobe than in Nur-Sultan city. Despite this, IVF success in Aktobe is higher (38%) than in Nur-Sultan (12%), and pregnancy rate was statistically significantly higher among women who lived in Aktobe city (<0.001). Given the lower number of oocytes retrieved and the number of embryos implanted in Aktobe, one may expect the pregnancy rate to be lower in Aktobe in comparison to Nur-Sultan. A possible explanation for our finding is that the National Research Center for Mother and Child Health in Nur-Sultan has more complicated cases with less probability of pregnancy and expected lower results. On the other hand, Aktobe clinic may be pre-selecting women with better possible outcomes (fewer complications, less infertility duration time, etc.). Nevertheless, in our study, we confirmed that money source (governmental or out-of-pocket) did not have any influence on IVF outcome. 

As would be expected, data from this study revealed higher pregnancy rates for patients who had multiple embryos transferred, relative to single embryo transfer (SET) patients. Therefore, it is reasonable to predict that IVF clinics following the recent standards may be expected to have lower pregnancy rates. This prediction is supported by recently published data from regional registries of the USA, Canada, the UK, Australia/New Zealand (combined), Latin America (as a block), and Japanese governments and/or specialty societies which reveal trends of lower pregnancy rates [28]. However, SET is associated with better health outcomes for the woman and the developing fetus at the conclusion of the pregnancy. Further, research indicates that if patients who are unsuccessful with the initial SET then undergo a second SET, the total number of women who attain a pregnancy actually exceeds the number of women who attain a pregnancy when two embryos are transferred [29].

Study strength and limitations. The main study limitation is the potential presence of nonresponse bias associated with the low response rate. We did not have data on demographic, clinical and psychological characteristics of non-respondents to make informed conclusion of whether the study participants were systematically different from non-respondents. We could speculate that non-respondents had worse reproduction prognosis, thus they were likely depressed, stressed or anxious about IVF and unwilling to participate in the study. Another limitation is low statistical power given the small sample size. Post hoc statistical power analysis revealed that the statistical power was lower than the recommended 80% level in all psychometrical scales: CES-D scale had the lowest power (24.2%); STAI-S—60%, STAI-T 48%, and FPI subscales had statistical power between 60% to 70%. Increasing the number of patients might provide statistically significant results. Despite low statistical power, we found statistically significant associations between IVF outcome and anxiety, state and trait, levels in the multivariable analysis. It is possible that participants with worse reproductive prognosis were likely in distress given their previous unsuccessful IVF attempts. Thus, determining temporal association between anxiety (as exposure) and IVF outcome (as outcome) is challenging. This is another study limitation. Since this study also included patients with repeated IVF cycles, it would be advised to conduct a future study including only first-time IVF patients to confirm the temporal association. A strength of this study is that the evaluation of stress, depression and anxiety in patients undergoing IVF in the Kazakhstan ART clinics was performed in relation to the procedure outcomes. 

Clinical implication. Although neither the International Committee for Monitoring Assisted Reproductive Technologies, the American Society for Reproductive Medicine, nor the European Society for Human Reproduction and Embryology have formal requirements for psychological counseling of infertile couples, there is acknowledgement that incorporating psychological interventions into routine practice in IVF clinics is beneficial [11]. We suggest that the results of this study and understanding of the presence and level of stress anxiety and depression in Kazakhstani women with infertility undergoing IVF treatment are reasons to implement psychological support in ART clinics. The support should be provided by a certified specialist on a regular basis before and during the treatment. Routinely incorporating the use of the questionnaires used in this study (or similar tools) into the clinical practice of ART clinics may be useful in detecting and ameliorating depression and anxiety in infertility patients.

## 5. Conclusions

A diagnosis of infertility could become a serious challenge for couples. It remains a major health care problem. A large proportion of patients attending public IVF clinics experience depressive symptoms and they should be counseled and supported through the treatment course. It has been well documented before and supported by the results of our study that infertility causes stress, depression, and anxiety, although the degree of these experiences may vary. The multiple negative binomial regression analysis in this study shows that state and trait anxiety are associated with lower chances of pregnancy in public IVF clinics. Further research is needed to clarify whether this association is confounded by other factors (such as smoking, embryos quality, and others) and patients’ awareness about their reproductive prognosis. The impact of stress on IVF outcome remains unclear. However, it is clear that psychological interventions for women with infertility has the potential to decrease anxiety and depression. Further research may clarify whether efforts to decrease depression and anxiety impact treatment outcomes.

## Figures and Tables

**Table 1:** Demographic, past clinical and in vitro fertilization (IVF) treatment characteristics of the study participants.

Variable	Total, *n* = 142	Pregnant, *n* = 35	Not Pregnant, *n* = 83	*p*-Value	Unknown, *n* = 24
**Demographic variables**					
Age (years)	33.9 ± 4.9	32.9 ± 4.7	34.3 ± 4.9	0.18	33.9 ± 5.4
Missing data = 3.5%					
BMI					
Underweight	10 (7.3%)	7 (20.0%)	1 (1.3%)	<0.01	2 (8.7%)
Normal	76 (55.5%)	18 (51.4%)	46 (58.2%)		12 (52.2%)
Overweight/Obese	51 (37.2%)	10 (28.6%)	32 (40.5%)		9 (39.1%)
Missing data = 3.5%					
Location					
Nur-Sultan	75 (52.8%)	9 (25.7%)	52 (62.6%)	<0.001	14 (58.3%)
Aktobe	67 (47.2%)	26 (74.3%)	31 (37.4%)		10 (41.7%)
Missing data = 0%					
**Past and current medical history of infertility**					
Comorbidity					
Yes	83 (58.4%)	8 (22.9%)	59 (71.1%)	<0.001	16 (66.7%)
No	59 (41.6%)	27 (77.1%)	24 (28.9%)		8 (33.3%)
Missing data = 0%					
Infertility duration (years)					
Mean ± SD	6.0 ± 3.5	6.4 ± 2.9	6.0 ± 3.9	0.57	5.5 ± 3.2
Median (IQR)	6 (3–8)	7 (4.5–8)	5 (3–8)		5 (3–8.5)
Missing data = 4.9%					
Number of previous IVF cycles					
None	106 (75.2%)	30 (85.7%)	56 (67.5%)	0.05	20 (87.0%)
One	25 (17.7%)	2 (5.7%)	20 (24.1%)		3 (13.0%)
2 or more	10 (7.1%)	3 (8.6%)	7 (8.4%)		0 (0%)
Missing data = 0.7%					
**IVF treatment characteristics**					
Number of oocytes retrieved					
Mean ± SD	8.1 ± 7.2	8.3 ± 5.0	7.9 ± 8.0	0.11	8.4 ± 7.6
Median (IQR)	6 (3–11)	9 (4–11)	5 (2–10)		8 (3–9)
Missing data = 11.3%					
Number of embryos transferred					
Mean ± SD	1.4 ± 1.1	1.8 ± 1.2	1.2 ± 1.1	0.01	1.5 ± 0.7
Median (IQR)	1 (1–2)	2 (1–2)	1 (1–2)		2 (1–2)
Missing data = 14.1%					

**Table 2:** Depression, stress and anxiety scale scores between pregnant and not pregnant women.

Scales	Total, *n* = 142	Pregnant, *n* = 35	Not Pregnant, *n* = 83	*p*-Value	Unknown, *n* = 24
CES-D score, mean ± SD	18.3 ± 9.6	21.9 ± 8.9	16.8 ± 9.8	0.01	18.4 ± 9.0
Categorized CES-D score, *n* (%)					
At risk for clinical depression (≥16)	84 (59.1%)	28 (80.0%)	43 (51.8%)		13 (54.2%)
Missing data = 0%					
FPI scale					
Social concern, mean ± SD	29.5 ± 6.6	30.4 ± 7.1	28.9 ± 6.7	0.27	30.1 ± 5.0
Sexual concern, mean ± SD	20.4 ± 6.8	20.0 ± 7.5	20.1 ± 6.1	0.89	22.2 ± 7.7
Relationship concern, mean ± SD	28.0 ± 6.6	26.5 ± 6.3	28.2 ± 6.7	0.20	29.4 ± 6.6
Need for parenthood, mean ± SD	43.5 ± 9.1	45.9 ± 8.5	42.5 ± 9.5	0.07	43.3 ± 7.9
Rejection of childfree lifestyle, mean ± SD	32.0 ± 6.2	31.7 ± 5.8	31.9 ± 6.6	0.85	32.4 ± 5.9
Global stress, mean ± SD	153.3 ± 18.4	154.6 ± 17.0	151.7 ± 19.5	0.45	157.4 ± 16.4
Missing data = 0.7%					
STAI State, mean ± SD	41.9 ± 11.4	42.8 ± 11.4	42.5 ± 11.4	0.89	38.4 ± 10.8
STAI Trait, mean ± SD	44.1 ± 8.4	45.4 ± 6.5	44.2 ± 9.1	0.54	42.1 ± 8.0
Missing data = 0%					

**Table 3:** Simple and multiple negative binomial regression analyses of psychological factors predicting IVF clinical pregnancy.

Scales	RR_Crude_ (95% CI)	^&^ RR_Adj_ (95% CI)
CES-D score	1.04 (1.00–1.07) *	0.99 (0.95–1.04)
FPI scale		
Social concern	1.02 (0.97–1.08)	0.99 (0.93–1.04)
Sexual concern	0.99 (0.95–1.06)	0.97 (0.92–1.02)
Relationship concern	0.97 (0.93–1.02)	0.96 (0.91–1.01)
Need for parenthood	1.03 (0.99–1.07)	1.03 (0.99–1.07)
Rejection of childfree lifestyle	0.99 (0.94–1.05)	1.00 (0.94–1.07)
Global stress	1.01 (0.99–1.02)	1.00 (0.98–1.02)
STAI State	1.00 (0.97–1.03)	0.95 (0.91–0.99) *
STAI Trait	1.01 (0.97–1.05)	0.92 (0.86–0.99) *

RR—relative risk. ^&^ The models were adjusted for women’s BMI, location, comorbidity, and number of previous IVF cycles. * *p* < 0.05, statistically significant result.

## Data Availability

The data are uploaded to Open Science Framework: osf.io/2bknw(accessed on 26 February 2021).

## Supplementary Materials

The following are available online at https://www.mdpi.com/2077-0383/10/5/937/s1, Table S1: additional data on demographic characteristics of the study participants, Table S2: additional data on past and current medical history of infertility of the study participants, Table S3: additional data on IVF treatment characteristics of the study participants, Table S4: regional differences in socio-demographic, clinical characteristics of the study participants, Table S5: regional difference in depression, stress, and anxiety scales’ scores among the study participants.
