# Supplementary material for: The Effect of Stress, Anxiety and Depression on In Vitro Fertilization Outcome in Kazakhstani Public Clinical Setting: A Cross-Sectional Study
Source: J Clin Med. 2021 Mar 1;10(5):937. doi: 10.3390/jcm10050937 (PMC7975982; doi:10.3390/jcm10050937)
Supplement: Supplementary file 1 [file jcm-10-00937-s001.pdf]

Supplementary Tables

Supplementary Table 1. Additional data on demographic characteristics of the study participants

Variable	Total, N=142	Pregnant, n=35	Not pregnant, n=83	p-value	Unknown, n=24
Education level					
ISCED 3	3 (2.1%)	1 (2.9%)	1 (1.2%)	0.25	1 (4.2%)
ISCED 4	48 (34.3%)	9 (25.7%)	32 (39.5%)		7 (29.2%)
ISCED 5	26 (18.6%)	4 (11.4%)	14 (17.3%)		8 (33.3%)
ISCED 6	63 (45.0%)	21 (60.0%)	34 (42.0%)		8 (33.3%)
Missing data=1.4%					
Type of payment					
State-funded	85 (59.9%)	25 (71.4%)	44 (53.0%)	0.06	16 (66.7%)
Self-paid	57 (40.1%)	10 (28.6%)	39 (47.0%)		8 (33.3%)
Missing data=0%					

Supplementary Table 2. Additional data on past and current medical history of infertility of the study participants.

Variable	Total, N=142	Pregnant, n=35	Not pregnant, n=83	p-value	Unknown, n=24
Number of previous deliveries					
None	106 (74.6%)	30 (85.7%)	59 (71.1%)	0.09	17 (70.8%)
One or more	36 (25.4%)	5 (14.3%)	24 (28.9%)		7 (29.2%)
Missing data=0%					
Number of previous miscarriages					
None	127 (89.4%)	33 (94.3%)	72 (86.7%)	0.34	22 (91.7%)
One or more	15 (10.6%)	2 (5.7%)	11 (13.3%)		2 (8.3%)
Missing data=0%					
Number of previous intentional pregnancy interruptions					
None	125 (88.0%)	33 (94.3%)	73 (88.0%)	0.51	19 (79.2%)
One or more	17 (12.0%)	2 (5.7%)	10 (12.0%)		5 (20.8%)
Missing data=0%					
Cause of infertility					
Female	57 (40.4%)	19 (54.3%)	29 (35.4%)	0.12	9 (37.5%)
Male	8 (5.7%)	2 (5.7%)	5 (6.1%)		1 (4.2%)
Mixed	76 (53.9%)	14 (40.0%)	48 (58.5%)		14 (58.3%)
Missing data=0.7%					

Supplementary Table 3. Additional data on IVF treatment characteristics of the study participants.

Variable	Total, N=142	Pregnant, n=35	Not pregnant, n=83	p-value	Unknown, n=24
Used protocol					
Classic-long	5 (3.7%)	2 (5.9%)	2 (2.4%)	0.05	1 (5.6%)
Classic-short	122 (90.4%)	28 (82.3%)	77 (92.8%)		17 (94.4%)
Non-classic – natural cycle	2 (1.5%)	0 (0%)	2 (2.4%)		0 (0%)
Non-classic – ultrashort	5 (3.7%)	4 (11.8%)	1 (1.2%)		0 (0%)
Non-classic – stimulated in luteal phase	1 (0.7%)	0 (0%)	1 (1.2%)		0 (0%)
Missing data=4.9%					
Fertilization rate, %					
Mean±SD	88±22	94±8	85±24	0.63	100±0
Median (IQR)	96 (80-100)	97 (92-99)	93 (80-100)		100
Missing data=65.5%					

Supplementary Table 4. Regional differences in socio-demographic, clinical characteristics of the study participants.

Variable	Nur-Sultan, n=75	Aktobe, n=67	p-value
Age(years)	32.6±4.7	35.4±4.7	<0.001
BMI			
Underweight	5 (6.8%)	5 (7.9%)	0.80
Normal	43 (58.1%)	33 (52.4%)	
Overweight/Obese	26 (35.1%)	25 (39.7%)	
Education level			
ISCED 3	3 (4.1%)	0 (0%)	<0.001
ISCED 4	42 (57.5%)	6 (9.0%)	
ISCED 5	17 (23.3%)	9 (13.4%)	
ISCED 6	11 (15.1%)	52 (77.6%)	
Cause of infertility			
Female	16 (21.3%)	41 (62.1%)	<0.001
Male	6 (8.0%)	2 (3.0%)	
Mixed	53 (70.7%)	23 (34.9%)	
Number of oocytes retrieved			
Mean±SD	10.1±8.1	5.8±5.1	<0.01
Median (IQR)	8 (4-14)	4 (1-10)	
Number of embryos transferred			
Mean±SD	1.6±0.8	1.3±1.4	<0.01
Median (IQR)	2 (1-2)	(0-2)	

Supplementary Table 5. Regional difference in depression, stress, and anxiety scales' scores among the study participants.

Scales	Nur-Sultan, n=75	Aktobe, n=67	p-value
Continuous CES-D score	13.1±8.9	24.2±6.7	<0.001
Categorized CES-D score			
At risk for clinical depression (≥ 16)	23 (30.7%)	61 (91.0%)	<0.001
FPI scale			
Social concern	27.5±7.1	31.7±5.2	<0.01
Sexual concern	18.2±6.4	22.9±6.4	<0.001
Relationship concern	26.0±6.8	30.2±5.7	<0.001
Need for parenthood	44.5±8.4	42.4±9.7	0.16
Rejection of childfree lifestyle	32.1±6.8	31.8±5.6	0.74
Global stress	148.4±20.4	158.9±14.1	<0.001
STAI State	36.5±10.7	48.0±8.8	<0.001
STAI Trait	40.1±8.9	48.7±4.7	<0.001
